# AND Gates as Sources of Biological Nonlinearity: Relevance to Genetic Dominance

**DOI:** 10.1002/adbi.70168

**Published:** 2026-10-26

**Authors:** Anis Toussirt, Reiner A. Veitia

**Affiliations:** ^1^ Institut Jacques Monod, Université Paris Cité CNRS Paris France; ^2^ Institut De Biologie François Jacob, Université Paris‐Saclay Commissariat à l’Énergie Atomique Fontenay‐aux‐Roses France

**Keywords:** AND‐gating, gene dosage, haploinsufficiency, sigmoidal response, transcriptional nonlinearity, transcriptional synergy, transcription factor

## Abstract

AND‐gating is a mechanism capable of generating nonlinear biological responses because a productive output requires the joint satisfaction of several necessary molecular events. AND gates are considered here in the context of gene expression. Three minimal architectures are discussed, parallel AND gates, kinetic AND gates, and a serial arrangement of AND gates. In a parallel AND gate, output depends on the simultaneous engagement of several modules. In a kinetic AND gate, output requires the completion of several events within a finite time window. In a serial arrangement, the output of one gate becomes the input of the next. These architectures can generate S‐shaped responses even when the elementary processes are non‐cooperative. Although transcription is our main focus, the same logic applies to other processes. The sigmoidal responses produced by these AND gates provide a phenomenological explanation for how a reduction in the abundance or activity of a dosage‐sensitive molecule can generate more‐than‐proportional downstream effects, a hallmark of haploinsufficiency (HI), a form of dominance. The same AND‐gate logic also sheds light on dominant‐negative effects, where a mutant protein interferes with its wild‐type counterpart. These models illustrate how AND gate architectures can contribute to biological threshold generation and to nonlinear genotype–phenotype relationships, which underlie dominance.

## Graphical abstract

Biological switches often demand that several molecular conditions hold simultaneously. The authors show that such AND‐gating generates S‐shaped responses even when every underlying step is gradual and non‐cooperative. Near the transition, halving a regulator abundance produces a far greater drop in output, offering a simple explanation for dominance and gene dosage sensitivity.

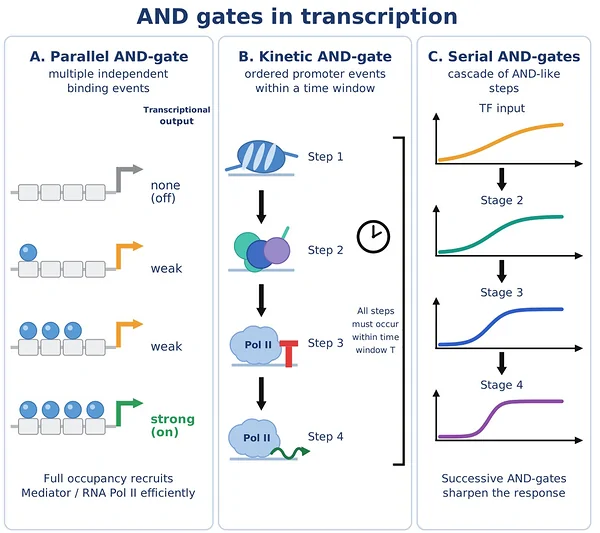

## 1 Introduction

Genetic dominance represents a nonlinear genotype–phenotype mapping because the phenotype of the heterozygote does not lie midway between those of the two homozygous states [ 1]. Haploinsufficiency (HI) is a form of dominance in which about half the normal amount of a gene product (due, for instance, to heterozygous loss‐of‐function mutations) is insufficient to produce a normal phenotype. Dominant‐negative (DN) mutations, in turn, are pathogenic variants leading to an altered gene product that interferes with the function of the wild‐type one [ 2]. Pathogenic variants in many genes encoding transcription factors (TFs) often lead to HI or DN effects [ 3]. As an example in the context of HI, experimental variation of the TF SOX9, which is haploinsufficient, shows that even modest reductions in protein levels can produce more‐than‐proportional reductions in transcriptional output for a subset of target genes [4].

Transcriptional initiation requires the completion of multiple molecular events, including TF recruitment to cis‐regulatory sequences, cofactor recruitment, enhancer–promoter communication, preinitiation complex assembly, promoter opening, promoter escape, pause release, and early elongation [5]. A promoter may therefore fire efficiently only when such necessary conditions are satisfied [6, 7, 8, 9], which underlies an AND gate architecture. This term is borrowed from digital logic: an AND gate is a switch that turns ON only when every one of its inputs is ON. Below, the completion of the process of promoter complex assembly and initial transcription is called ‘promoter firing’ for simplicity.

Here, we discuss three minimal implementations of AND gates. The first is a parallel AND gate. In general terms, if the required events are independent, the probability of productive firing can be represented as the product of their individual success probabilities. When the individual probabilities increase gradually and noncooperatively, and saturate as a function of the regulatory input, their product can generate a sigmoid‐like input–output response ([10] and references therein). The second model is a kinetic AND gate, in which productive output requires several time‐limited molecular events. The third is a serial arrangement of AND gates. In a broader context, the same logic applies to other molecular processes governed by conjunctions of requirements.

The general models discussed here do not attempt to reproduce the full complexity of any specific system. They simply illustrate how multiple requirements can generate sigmoidal responses and how, when a molecular process operates near the transition zone of an AND‐gated input–output curve, a reduction in input can produce a more‐than‐proportional decrease in output and potentially contribute to dominance and especially to HI.

## 2 Parallel AND Gate

Transcriptional synergy is a specific example of AND‐gating. Unlike classical cooperative DNA binding, in which binding of one TF molecule increases the affinity or residence time of another [11], independently bound TFs can still generate a nonlinear output if productive activation depends on their joint recruitment of a multivalent macromolecular platform [12, 13]. Synergy does not exclude cooperativity; loosely speaking, it acts downstream, when the TF molecules are already bound to DNA.

In thermodynamic terms, each activator–platform contact contributes a favorable binding free energy. These contributions are approximately additive at the free‐energy level, whereas their effects on association strength are multiplicative because ΔG° ═ –RT ln K_a_, where K_a_ is an association constant. Thus, several individually weak contacts can collectively produce strong recruitment of the transcriptional machinery. When affinity is expressed using dissociation constants, the combined interactions strongly decrease the effective dissociation constant, provided that multivalent binding does not incur a substantial energetic penalty, which is a highly idealized but convenient simplifying assumption for theoretical purposes [13]. To model transcriptional synergy as an example of an AND gate, consider that a DNA element contains n independent binding sites for a TF A. In this context, BA_i_ denotes a DNA molecule carrying i bound molecules of A (i ═ 0, …, n). The system therefore encompasses states B, BA, BA_2_, …, BA_i_, …, BA_n_. Each site is assumed to bind A independently. Thus, binding to one site does not alter the affinity of A for the others. The fractional occupancy of a single site is:

(1) $$\theta =\frac{\left[A\right]}{K_{i}+\left[A\right]}$$

where [A] is the free concentration of A and K_i_ is the dissociation constant for the site i. Because the n sites are independent, the probability that all sites are occupied is the product of the individual occupancies. For simplicity, we assume below that all the sites have the same dissociation constant (K_i_ ═ K). In this condition, the fraction of promoters in the fully occupied state is:

(2) $$\theta^{n}={\left(\frac{\left[A\right]}{K+\left[A\right]}\right)}^{n}$$

This is a Hill‐like equation and describes an S‐shaped curve for n > 1. The classical Hill equation (i.e., H  ═ [A]^*nH*^ /(K^*nH*^  +  [A]^*nH*^)) is used to describe a sigmoidal response in phenomenological terms [14]. The parameter nH defines the steepness of the Hill sigmoid. If, by virtue of synergy, the fully occupied promoter is the state that efficiently drives transcription, the concentration of transcriptionally competent promoters [BA_n_] is proportional to θ^n^. The transcriptional output, dominated by the fully occupied promoter state, is therefore S‐shaped without requiring cooperative DNA binding. Consistently, a sigmoidal response to increasing activator concentration has been documented as a result, at least in part, of transcriptional synergy [15], and mathematical model‐fitting to data has provided evidence for a multiplicative effect in the recruitment of the transcriptional machinery [16]. The interest of the relation [BA_n_] ∝ θ^n^ lies in its utter simplicity while still accounting for a Hill‐like behavior over its transition region, where a modest change in (free) [A] can produce a more‐than‐proportional change in transcriptional output. To give a concrete sense of scale, for n ═ 4: if the system operates near saturation (e.g. [A] ═ 9K, θ ═ 0.9), halving [A] leads only to a ∼1.5‐fold drop of the fully‐occupied fraction θ^4^. If instead the system operates near half‐occupancy of each individual site ([A] ═ K, θ ═ 0.5), the same halving of [A] leads to a ∼5‐fold drop of θ^4^. The same architecture can therefore be dosage‐sensitive or not depending on where along the curve the system happens to sit. This is a point developed quantitatively below.

There are two main ways to obtain a quantitative idea of the steepness of the Hill‐like curves. The first is to estimate an apparent Hill coefficient, nH (n_fit_ from now on) using least‐squares fitting to a standard Hill function. Because this estimate is based on a regression, it depends on the input range covered by the fit. A simpler crude estimate is given by the relation $n_{10-90}=\ln\left(81\right)/\ln\left({\left[A\right]}_{90}/{\left[A\right]}_{10}\right)$, where [A]_10_ and [A]_90_ are the inputs giving 10% and 90% of the maximal response. This measure returns the exact Hill coefficient when applied to a Hill function. In the case of a Hill‐like curve, if we take θ^4^ versus [A] (for K ═ 0.1), a fit over an operating range of say, 0%–90% of the maximal response yields an n_fit_ ≈ 1.21 and n_10 − 90_ ≈ 1.30 (Figure 1). The Hill function describes the curve well over this range (R^2^ ≈ 0.99), so the modest value of *n_fit_* reflects an actually shallow response. That said, a system does not have to reach 90% of its maximum capacity, which has an interesting consequence. For example, if the same promoter of Figure 1 operates over the range 0 ≤ [A] ≤ 1 units of [A], where the response reaches only ∼68% of its maximum, a Hill fit over that window yields a higher *n_fit_* ≈ 1.37. This example shows that the estimated steepness depends on the operating range considered for the fitting.

**FIGURE 1:**
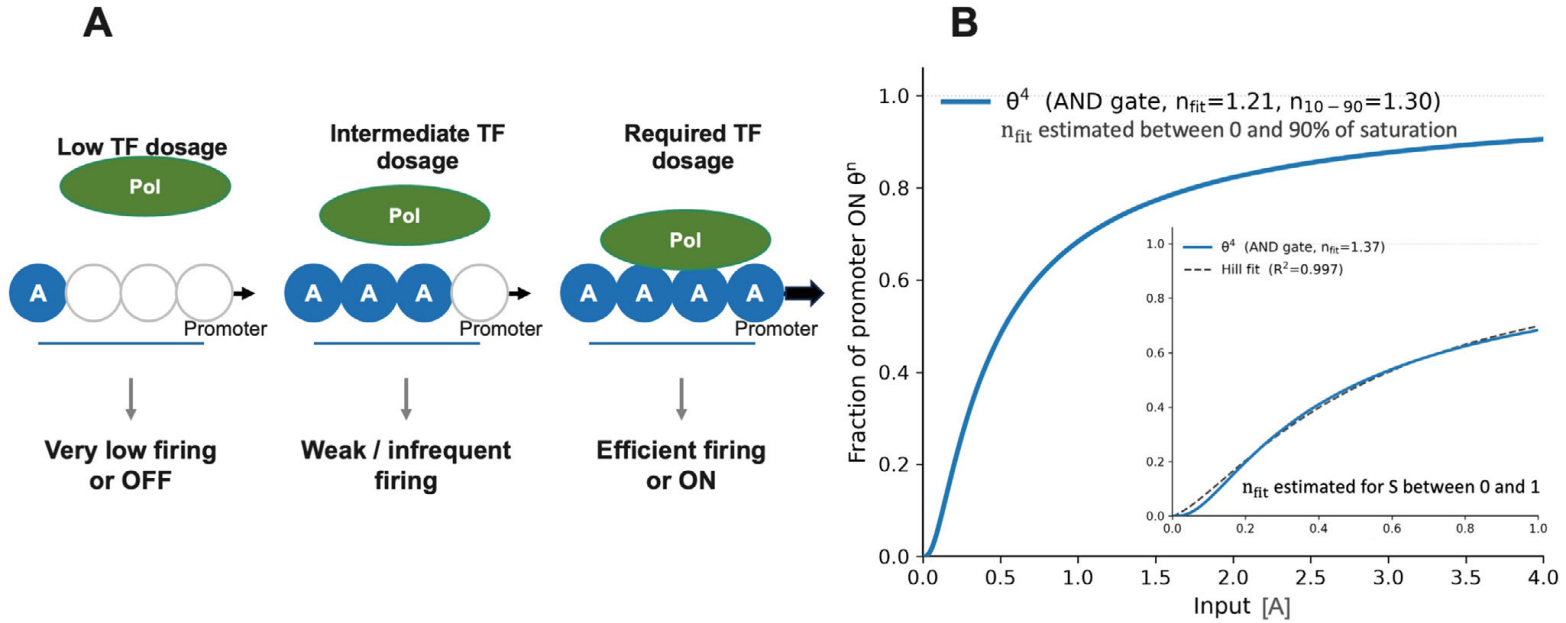
Transcriptional synergy as a parallel AND gate. (A) Representation of a promoter containing several independently occupied sites. Three representative regimes are shown. Each site responds gradually to transcription‐factor concentration, but efficient recruitment of a molecular platform (e.g., Mediator complex and/or RNA polymerase II) requires simultaneous occupancy of all sites in this particular case. Incomplete occupancy produces little or no productive transcription, whereas full occupancy permits efficient promoter complex assembly/firing. (B) For illustrative purposes, the curve θ^n^ versus [A] uses *K* ═ 0.1 and *n* ═ 4, corresponding to four required occupancy events. Apparent steepness estimated as *n_fit_* ═ 1.21 and n_10__−__90_ ═ 1.30, both far below n. Inset: for an operating range spanning 0 ≤ [A] ≤ 1 units of [A], *n_fit_* ═ 1.37.

The previous example suggests that both n_fit_ and n_10 − 90_ are similar and lower than n. To confirm that *n*_10 − 90_ can be used to obtain an estimate of *n_fit_* we produced 375 Hill‐like functions (n from 2 to 1000 and K from 0.01 to 10), fitted them to Hill equations to obtain *n_fit_* to compare with *n*_10 − 90_. The ratio *n*_10 − 90_/*n_fit_* was <1.08, showing the strong similarity of both measures. Both *n_fit_* and *n*_10 − 90_ are independent of K for the 0 ‐ 90% fitting range used here because θ^n^ depends on [A] and K only through the ratio [A]/K. In addition, *n*_10 − 90_ rises with n toward a finite ceiling. At n ═ 1000, the computed value is 1.423. In closed form *n*_10 − 90_═ ln(81)/ln[^n^√9 · (1 − ^n^√0.1)/(1 − ^n^√0.9)]. As n → ∞, *n*_10 − 90_ approaches a fixed ceiling of ln(81)/ln[ln(10)/ln(10/9)] ≈ 1.4247. This is in agreement with what we observe in the simulations above. This ceiling lies far below a more general theoretical bound. At thermodynamic equilibrium, the sharpness achievable by a system with n binding sites cannot exceed a Hill coefficient of n, a result known as the “Hopfield barrier” [17], which corresponds to the limit of infinite cooperativity [13] and references therein. Overall, AND‐gating by independent sites reliably produces a response that is steeper than hyperbolic (*n_fit_* > 1) but never approaches the steep, switch‐like behavior that the number of sites involved, n, might suggest (*n_fit_* << n).

Coming back to genetics, in the case of a heterozygous deletion of a TF‐encoding gene, one expects only two values of A (for the initial and halved conditions) and two corresponding promoter firing probabilities for each target, not the continuous curve. The continuous curve in Figure 1 is not a trajectory for any single gene. It represents the underlying dose‐response relationship for target promoters with comparable K values, evaluated across possible operating points. Plotting it this way lets the two‐point, before/after comparison relevant to a given heterozygous loss‐of‐function mutation be read off at whichever operating point that gene happens to sit.

The existence of synergy can also help explain, at least in part, the effect of some DN mutations in TFs. For instance, a transactivation‐domain‐deleted TF can keep its DNA‐binding capacity intact, so it competes with the wild‐type form for the same binding sites. It does not need to ‘win’ the competition, it only needs to occupy one slot to make the AND condition fail for that promoter. At equimolar wild‐type/mutant expression, this failure rate can be sharp. Loosely speaking, only (1/2)^n^ of promoters end up fully loaded with n active monomers (i.e., θ^n^ where θ  ═  1/2). Thus, a two‐site promoter for equal WT/mutant abundances and affinities is predicted to retain just 25% of wild‐type output, a three‐site promoter 12.5%, and so on. Of course, these values should be taken as limiting cases because many TFs do not act alone (often as dimers), may retain residual transactivation, or the deletion could modify the affinity for DNA. In the realm of human genetics, C‐terminal truncating mutations of PAX6 that remove most of the C‐terminal transactivation domain while preserving the paired domain and homeodomain intact produce a dominant‐negative effect leading to aniridia‐related phenotypes. In addition, because the deletion also relieves an autoinhibitory constraint on DNA binding, the truncated protein binds target sites more avidly than wild‐type [18]. Nature uses a similar strategy in a physiological context: Stat5 and Stat6 achieve a similar outcome through regulated proteolysis that removes the C‐terminal transactivation domain, converting a fraction of the DNA‐binding‐competent, dimerization‐competent pool of protein into AND‐gate‐breakers [19].

Two recent examples of synergistic systems involve p300 and Mediator. The former has been proposed to act as an integrator of combinatorial TF inputs, because individual TF‐p300 interactions are insufficient to drive transcriptional activation on their own but become effective when they act together [20]. This provides a typical example of “Velcro‐like” recruitment of a protein platform. A similar phenomenon has been documented for Mediator using single‐molecule fluorescence studies in yeast. Indeed, Mediator occupancy increases nonlinearly with activator concentration owing to multiple DNA‐bound activators that engage it simultaneously [21].

As already mentioned, the site‐occupancy calculation above (θ^n^) is a special case of the more general AND gate formulation, obtained by setting K_i_ ═ K for every required event. We show the general form below to cover cases where the n required conditions are not identical binding events. Let us assume that productive initiation requires the engagement of n necessary promoter modules (for example, occupancy of multiple TF sites, not necessarily by the same TFs). Let S denote the regulatory input, a positive dose in arbitrary units. We write a single S for readability, but nothing below requires it: when the modules respond to different regulators, S is simply replaced by a module‐specific input S_i_. For each module i, p_i_(S) is the probability that module i is engaged at a given moment. If engagement is individually graded and saturating, we can write:

(3) $$p_{i}\left(S\right)=\frac{S}{K_{i}+\;S}$$

where K_i_ is a constant corresponding to the dose at which module i is half‐engaged.

As a minimal assumption, the probability that all modules are ‘simultaneously’ satisfied, leading to promoter firing, is the product:

(4) $$P_{\mathrm{fire}}\left(S\right)=\prod_{i=1}^{n}p_{i}\;\left(S\right)=\prod_{i=1}^{n}\frac{S}{K_{i}+S}\;$$

The multisite independent‐binding model and the more general AND‐gate formulation are equivalent when the modules are the binding sites. When different activators are involved (A, B, C, …), S is replaced by the dose of each activator.

## 3 Kinetic AND Gate

Now assume that productive initiation requires completion of n steps within a relevant time window T. Let k_i_(S) denote the rate at which step i is completed, where S is the regulatory input. If completion of step i is modeled as a Poisson process, the probability that this step occurs within time T is:

(5) $$p_{i}\left(S\right)=1-e^{-k_{i}\left(S\right)T}\;$$

### 3.1 Parallel Completion

Assume, for simplicity, that completion of each step is independent of the others given S, and that the steps can proceed in parallel, as in several racers running toward a finish line within a shared deadline. The probability that all required steps are completed within the time window is:

(6) $$P_{\mathrm{fire}}^{\mathrm{parallel}}\left(S\right)=\prod_{i=1}^{n}p_{i}\;\left(S\right)=\prod_{i=1}^{n}\left(1-e^{-k_{i}\left(S\right)T}\right)$$

If the rate of each step increases with the regulatory input in a saturating manner, for example:

(7) $$k_{i}\left(S\right)=k_{i}^{\max}\frac{S}{K_{i}+S}\;$$

then

(8) $$P_{\mathrm{fire}}^{\mathrm{parallel}}\left(S\right)=\prod_{i=1}^{n}\left[1-\exp\left(-k_{i}^{\max}T\frac{S}{K_{i}+S}\right)\right]\;$$

In this phenomenological AND‐gating model, increasing S raises the probability that each required event will occur within the time window  T. Consequently, the probability that all requirements are satisfied increases steeply over an intermediate input range before approaching a plateau (Figure 2). When different events depend on distinct regulatory inputs, the model can be generalized by assigning a separate input to each event.

**FIGURE 2:**
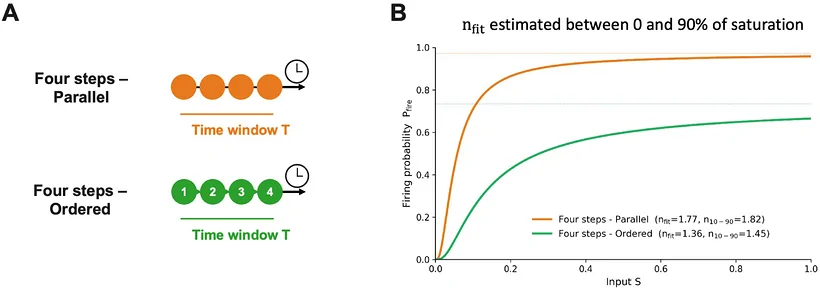
Multi‐step kinetic AND‐gate model. (A) Comparison of processes requiring four events that occur in parallel (orange), and four events that must occur in a strict sequence (green). Productive firing occurs only when all required events are completed before the end of the time window T. Parallel completion allows each event to use the entire time window, whereas ordered completion requires successive waiting times to fit within the same interval. (B) For *n* ═ 4 steps (*K_i_* ═ 0.1 and  $k_{i}^{\max}=\left[1;\;1;\;1;\;1\right]$, T ═ 5), the two architectures are compared. In the parallel model (orange curve), steps are independent and occur simultaneously, so the total probability is the product of individual completion probabilities. In the ordered model (green curve), there is a strict sequence of steps. For the parameter values used, the response is sigmoidal and the green curve is less steep and lower than the orange one.

### 3.2 Ordered Completion

The product above treats each step as if it had the full window T at its disposal. In an ordered process, step i+1 begins only after step i is completed, so the steps share a single time window where the sum of the successive waiting times τ_i_ is at most T. This ordered scenario resembles a relay, where each runner must wait for the baton before starting, so the same deadline is harder to meet.

With independent waiting times $\tau_{i}\;∼\;\mathrm{Exp}\left(k_{i}\left(S\right)\right)$ the total completion time is $W\left(S\right)=\sum_{i=1}^{n}\tau_{i}$ and productive firing requires W(S) ≤ T. The probability that all required steps are completed within the time window is:

(9) $$P_{\mathrm{fire}}^{\mathrm{ordered}}\;\left(S\right)=1-\sum_{i\;=1}^{n}\prod_{j\neq i}\frac{k_{j}\left(S\right)}{k_{j}\left(S\right)-k_{i}\left(S\right)}e^{-k_{i}\left(S\right)T}$$

Increasing S raises every rate and shortens the expected completion time, so P_fire_(S) can be sigmoidal over a broad range of relevant parameter values. This expression assumes pairwise distinct rates; in the special case of identical rates, the total completion time follows an Erlang distribution, and the corresponding cumulative distribution should be used. This ordered formulation is more constraining than the parallel one. Since the τ_i_ are non‐negative, $P_{\mathrm{fire}}^{\mathrm{ordered}}\left(S\right)\leq P_{\mathrm{fire}}^{\mathrm{parallel}}\left(S\right)$ for all S when using the same set of parameters for both models (Figure 2). This ordering holds in general. Parallel completion succeeds when max(τ_i_) ≤ T, and ordered completion succeeds when Στ_i_ ≤ T; because Στ_i_ ≤ T implies max(τ_i_) ≤ T, every realization in which the ordered process succeeds is one in which the parallel process also succeeds. Both architectures also yield apparent Hill coefficients substantially below n. As an illustration, for the parameters of Figure 2 (n  ═  4, K_i_ ═  0.1, $k_{i}^{\max}=\left[1;\;1;\;1;\;1\right]$, T  ═  5), where the rates are identical, and the Erlang form is therefore used, n_10 − 90_ ≈ 1.82 for the parallel gate and ≈ 1.45 for the ordered gate (both computed relative to the plateau of each curve).

The degree of sigmoidicity generated by the ordered kinetic AND gate depends on the overlap of the dynamic ranges of the individual steps. When the step‐rate curves are closely aligned, all steps accelerate over the same input range, giving the clearest sigmoid‐like transition; when they are poorly aligned, the overall response is broader and less ‘switch‐like’.

## 4 Serial Cascade of AND Gates

In this section we discuss an architecture that arises when one AND‐gated (or more generally, a sigmoidal) step becomes the input of the next. Here, a regulatory input enters at the head of the chain, and its influence is relayed and reshaped by every step downstream. This serial arrangement does not add the contributions of the individual steps. Instead, their local gains are multiplied, as already noted by [22] with a specific focus on MAPK signaling.

Let each stage j  ═  1, …, m be an elementary AND gate of the same form used above. Note that the index j runs over the successive stages of the cascade, whereas i was used above to index the modules within a single gate; each stage j therefore contains its own n_j_ required modules.

We write the AND‐gate j as:

(10) $$g_{j}\left(u\right)=A_{j}{\left(\frac{u}{K_{j}+u}\right)}^{n_{j}}\;$$

where *A_j_* sets the maximal output of stage *j* and simply rescales that stage so that the operating ranges of successive stages are comparable. Only the final output of the chain is interpreted as a firing probability. The cascade is then defined recursively as:

(11) $$\;x_{0}=\;S,\;\;x_{j}=g_{j}\left(x_{j-1}\right)$$

The final output is:

(12) $$P_{\mathrm{fire}}\left(S\right)=x_{m}=\left(g_{m}\circ \cdots \circ g_{1}\right)\;\left(S\right)\;$$

Thus, the input *S* enters only at the first stage, and every subsequent stage sees nothing but the output of the stage immediately before it. It is useful to define the ‘occupancy’ of stage *j* as:

(13) $$\;p_{j}=\frac{x_{j-1}}{K_{j}+x_{j-1}}\;$$

(14) $$\mathrm{so}\;\mathrm{that}\;x_{j}=A_{j}\cdot p_{j}^{\;n_{j}}\;$$

Because the cascade is a composition, the chain rule turns the logarithmic gain into a product of the gains at the various stages,

(15) $$\;\frac{\mathrm{dln}P_{\mathrm{fire}}}{\mathrm{dlnS}}=\prod_{j=1}^{m}R_{j},\;\;R_{j}=\frac{\mathrm{dln}x_{j}}{\mathrm{dln}x_{j-1}}=n_{j}\left(1-p_{j}\right)\;$$

In words, *R_j_* is the local fold‐change in a stage's output produced by a small fold‐change in its input; it plays the role of an elasticity in metabolic control analysis [23], and the overall gain is a response coefficient built as a product of these local sensitivities. Note that *R_j_* does not depend on *A_j_*: a multiplicative constant contributes an additive constant to the logarithm and therefore vanishes on differentiation, so the rescaling affects where the next stage operates but not how steeply it responds. *R_j_* ═ 1 means that the stage relays proportional changes unaltered, *R_j_* > 1 means it amplifies fold‐changes (a given fold‐change in input yields a larger fold‐change in output), and *R_j_* < 1 means it damps them. Because the stages are chained, the overall sensitivity of the cascade is the product of these local, stage‐by‐stage fold‐change gains.

Each stage can therefore sharpen or attenuate the signal it receives, and a cascade can compound this effect through the product of local gains (Figure 3). That said, multiplicative amplification is not automatic. Stages with R_j_ < 1 attenuate the signal and must be compensated by stages with sufficiently large R_j_ > 1. A plain hyperbolic stage, corresponding to n_j_ ═ 1, cannot amplify logarithmic gain because R_j_ ≤ 1. By contrast, a stage with n_j_ > 1 can amplify the signal, but only over the part of its dynamic range where:

(16) $$R_{j}=n_{j}\left(1-p_{j}\right)>1$$

**FIGURE 3:**
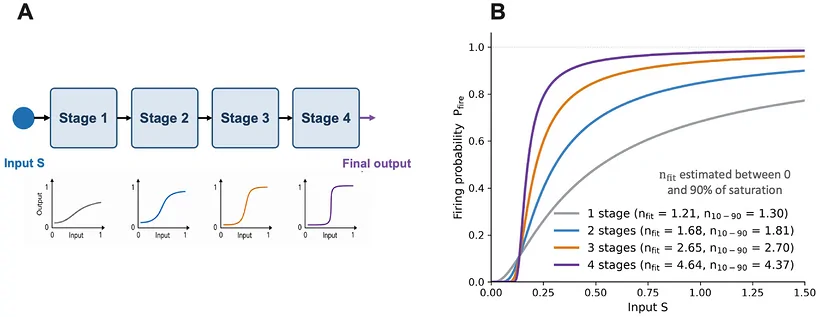
Series of AND gates. (A) Representation of a cascade in which the output of each AND‐gated stage becomes the input of the next. The small graphs below each stage show the output of the chain up to that stage, progressing to a sharp, switch‐like output after four stages. When the operating ranges of successive stages are appropriately aligned, the response becomes progressively steeper. Signal sharpening is not automatic and depends on the intrinsic nonlinearity and operating range of each stage. (B) Example of the progressive sharpening of the transcriptional response as a function of the number of stages. Each stage *j* is modeled as an elementary AND gate (here with *n_j_* ═  4 and *K_j_* ═  0.1) whose output has been rescaled so its plateau can reach about 1. Keeping successive operating ranges aligned and increasing the number of stages progressively steepens the input–output relationship.

Thus, serial amplification requires not only intrinsically thresholded stages, with n_j_ > 1, but also appropriate alignment of their operating ranges. That is, each downstream threshold K_j_ must be of the same order as the signal it receives: K_j_ ≲ $x_{j-1}^{\max}$.

## 5 Implications for Gene Dosage Sensitivity

From a general quantitative viewpoint, let $\varphi =P\left(S_{0}\right)/P\left(S_{0}/2\right)$ denote the factor by which the output decreases when the input is halved at an operating point S_0_. Because a two‐fold reduction is a finite perturbation, φ is governed by the logarithmic gain G(S)=dlnP/dlnS, averaged over the interval from S_0_/2 to S_0_, rather than by a single local slope or an apparent Hill coefficient.

For the serial AND‐gate model described above, this local gain can be written as:

(17) $$G\left(S\right)=\frac{d\ln P}{d\ln S}=\prod_{j}n_{j}\;\left(1-p_{j}\left(S\right)\right)$$

If we define the averaged logarithmic gain $\overset{-}{G}$(S_0_) over the interval [S_0_/2, S_0_] as:

(18) $$\overset{-}{G}\left(S_{0}\right)=\frac{1}{\ln2}\;\underset{S_{0}/2}{\int^{S_{0}}}G\left(S\right)\;\frac{\mathrm{dS}}{S}\;$$

then:

(19) $$\;\varphi =2^{\overset{-}{G}}\;\;\mathrm{or}\;\mathrm{equivalently}\;\overset{-}{G}\left(S_{0}\right)=\log_{2}\;\left[\frac{P\left(S_{0}\right)}{P\left(S_{0}/2\right)}\right]$$

The averaged exponent $\overset{-}{G}$ sets the response. If $\overset{-}{G}>1$ then φ > 2 and the output falls by more than two‐fold. Conversely, if $\overset{-}{G}<1$ then φ < 2 and the output is buffered relative to the input drop. $\overset{-}{G}$ is determined by the output at the two dosage states and requires no knowledge of the response between them (the integral representation above is simply a theoretical log‐input average of the local gain *G*(*S*) if we had access to the whole curve). To put this on a concrete scale: an averaged gain of $\overset{-}{G}$ = 1 corresponds to proportionality (a two‐fold drop in input causes exactly a two‐fold drop in output, φ = 2^1^); $\overset{-}{G}$ = 2 corresponds to a four‐fold drop in output (2^2^) for the same two‐fold drop in input and $\overset{-}{G}$ = 0.5 corresponds to only a ∼1.4‐fold drop in output (2^0.5^ ≈ 1.4, i.e. strong buffering). On the plateau of the sigmoid, $\overset{-}{G}\to 0$, and φ → 1. A process held in this saturated regime is insensitive to halving of its regulator. Conversely, halving the regulator of a target whose operating interval lies in a region where $\overset{-}{G}>1$, will produce a more‐than‐proportional loss (Figure 4). Under these assumptions, and when G(S) decreases over the interval [S_0_/2, S_0_], the effect of halving S_0_ can be bounded as follows:

(20) $$2^{G\left(S_{0}\right)}<\;\varphi <2^{G\left(\frac{S_{0}}{2}\right)}$$

**FIGURE 4:**
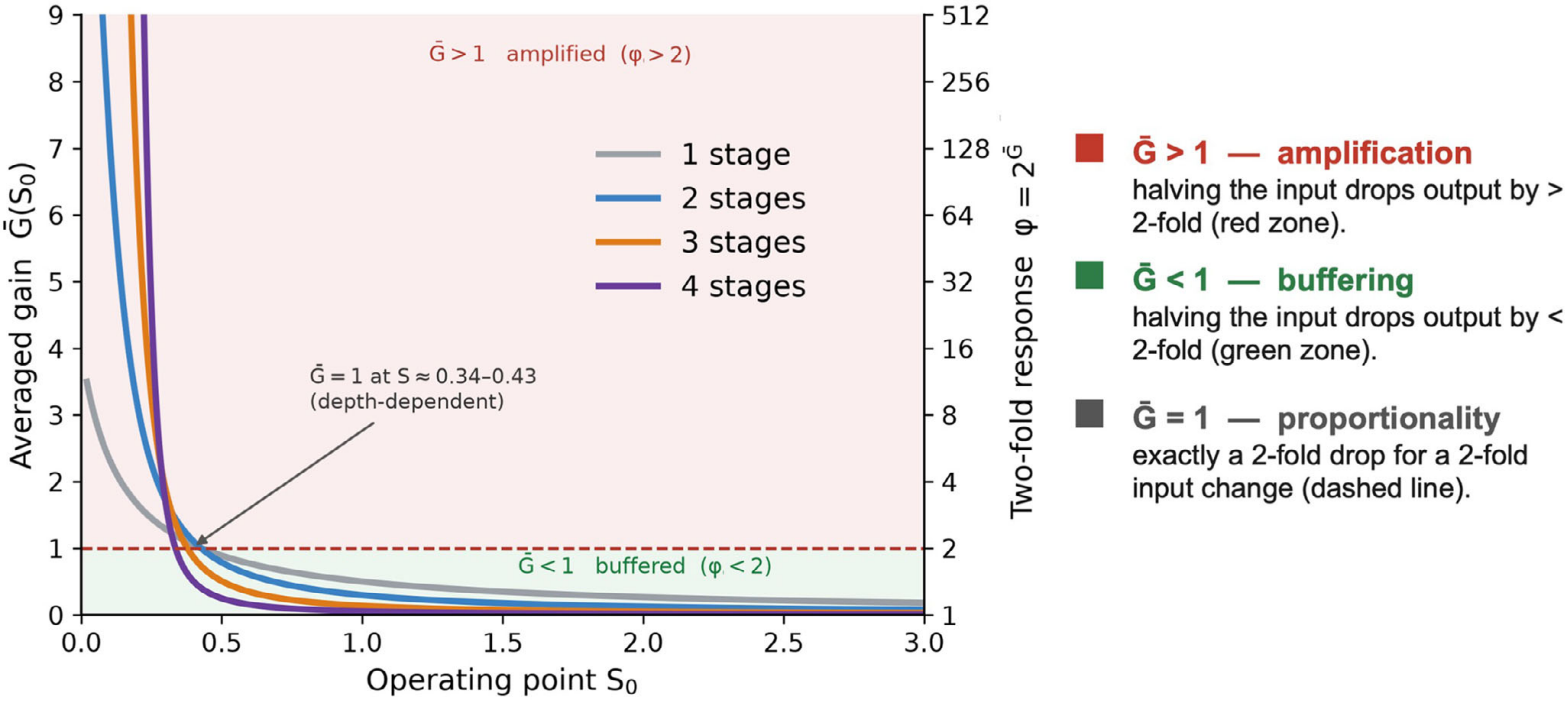
Dosage sensitivity evaluated at the operating point. The curves show 1‐ to 4‐stage cascades (n_j_ = 4, K_j_ = 0.1 per stage). The averaged logarithmic gain $\overset{-}{G}\left(S_{0}\right)$ (left axis) determines the factor $\varphi =2^{\overset{-}{G}}$ (right axis) by which the output falls when the input is halved (from S_0_ to S_0_/2). The threshold $\overset{-}{G}=1$ (red dashed line) separates the amplification regime ($\overset{-}{G}>1$, red area) where a two‐fold reduction in input results in a more‐than‐proportional decrease in output, from the buffering regime ($\overset{-}{G}<1$, green area), where the system is robust to dosage changes. Deeper cascades sharpen the transition between these two regulatory regimes.

This last inequality is not an additional assumption about dosage sensitivity. It is simply a convenient bound that follows when the logarithmic gain decreases over the interval, as in the elementary AND gate models considered here.

## 6 Discussion and Conclusions

The minimal models treated above show that mild sigmoidal biological responses can emerge from the conjunction (AND) of several graded, noncooperative requirements that are satisfied simultaneously. We have deliberately excluded cooperative DNA binding from the baseline models: any additional sharpness of the response observed beyond that provided by these AND gates comes from other sources. These sources can be classical cooperativity, even if cooperative TF binding itself is understood to go beyond classical pairwise interactions. For instance, higher‐order cooperativities among TFs and RNA polymerase have been shown to be necessary to explain transcriptional repression at a synthetic Drosophila enhancer [24]. A distinct route to sharp, threshold‐like transitions is provided by protein–DNA condensation. Indeed, DNA‐binding proteins and other factors of the transcription reaction can nucleate discrete condensates along DNA, generating thresholded gene expression [25, 26]. Other sources of nonlinearity that can have an impact on transcription include zero‐order ultrasensitivity and related phenomena in signaling cascades, sequestration or molecular titration, and bistability [27, 28, 29, 30, 31, 32]. AND‐gating and these mechanisms can have compounding effects.

A cautionary note: the conclusions drawn from the AND gate models remain contingent on simplifying assumptions, the most consequential being the independence of the sub‐events, which permits writing the firing probability as a product in the parallel AND model. The models also assume saturating elementary steps, quasi‐equilibrium deterministic binding in the parallel model and Poisson completion in the kinetic one, and often treat the fully occupied, completed, or assembled state as the sole productive state. Real systems may display partial activity, cooperative or antagonistic interactions, alternative pathways, spatial organization, feedback, stochastic events, and memory. These features may modulate the magnitude of the nonlinearity, but not the logical origin emphasized here.

The fact that AND‐gate‐like combinatorial logic can generate sigmoidal responses is well established. Previous treatments have shown how binding‐site affinities and pairwise cooperativities can be arranged to implement Boolean logic [33]. Classical thermodynamic modeling captures a wide range of such behavior [34]. In addition, the findings of Scholes et al. (2017) are related in spirit to the kinetic AND gate discussed in this paper [35]. Our simplified treatment asks how the steepness of the overall response is shaped by the number and properties of required steps.

Regarding dominance and HI in particular, we propose a quantitative criterion for distinguishing sensitive from buffered outputs applied to the AND gate framework but which can be applied to other systems as well. This measure translates the steepness of a response into a quantity directly relevant to HI: the averaged logarithmic gain $\overset{-}{G}$.

Before closing, it is worth noting that a similar AND‐gating logic applies to molecular processes other than transcription. In signaling, for example, activation of a downstream effector may require receptor engagement, adaptor recruitment, scaffold assembly, and the subsequent recruitment of enzymes catalyzing covalent protein modifications or RNA maturation, etc. Even if each requirement responds gradually to ligand concentration or another upstream input, pathway output can become sigmoidal. Examples from human genetics indicate that dosage sensitivity and dominant negative effects are not confined to transcriptional regulators. Heterozygous loss‐of‐function variants in RNA‐processing factors such as SON cause a human syndrome due to widespread splicing defects [36, 37], and variants in the spliceosomal factor PRPF31 cause autosomal‐dominant retinitis pigmentosa [38]. In sum, we argue that AND‐gated logic is not a peculiarity of transcriptional control but a recurring organizing principle that we have applied here to transcription.

## Funding

Anis Toussirt has a fellowship funded by PEPR Infertil‐Safe. RAV's salary is paid by Université Paris Cité.

## Conflicts of Interest

The authors declare no conflicts of interest.

## Artificial Intelligence Use

ChatGPT (OpenAI) and Claude (Anthropic) were used for language editing, improving clarity and accessibility of mathematical descriptions, checking mathematical derivations, generating illustrative numerical examples, assisting with figure design (conversion of sketches or drafts into final figure layouts) and helping to identify a few relevant references. All concepts, model assumptions, interpretations, conclusions and selection of examples were critically reviewed, revised, and approved by the authors.

## Data Availability

Data sharing not applicable to this article as no datasets were generated or analysed during the current study.
